# Impact of mixing coriander oil with goat feed on the chemical, microbiological and sensory characterizations of bio rayeb milk

**DOI:** 10.1038/s41598-023-38047-3

**Published:** 2023-07-11

**Authors:** Hagar S. Abd-Rabou, Hanem M. M. Mansour, O. H. Matloup, S. M. A. Sallam, M. A. Elazab

**Affiliations:** 1 Department of Food Technology, Arid Lands Cultivation Research Institute, The City of Scientific Research and Technological Applications (SRTA-City), Alexandria, 21934, Egypt; 2 Dairy Science Department, National Research Centre, 33 Bohouth St. Dokki, Giza, Egypt; 3 Department of Animal Production, Faculty of Agriculture, Alexandria University, El-Shatby, Alexandria, Egypt; 4 Department of Livestock Research, Arid Lands Cultivation Research Institute, City of Scientific Research and Technological Applications (SRTA-City), Alexandria, 21934 Egypt

**Keywords:** Biochemistry, Microbiology

## Abstract

This research aimed to investigate the properties of bio rayeb milk that results from goats fed on feed supplemented with different concentrations of coriander oil. The study design included a control treatment (C) and two coriander oil concentrations, a low level of (0.95%) T1 and a high level of (1.9%) T2. A probiotic starter culture, Direct Vat Set (DVS) of *lactobacillus delbrueckii* ssp*. bulgaricus* and *streptococcus salivarius* ssp*. thermophilus* in the ratio (1:1) was used to prepare bio rayeb. All treatments were stored at 4 °C for 2 weeks and analyzed on day one and at the end of storage. Results showed that the coagulation time during bio rayeb manufacturing remained consistent at almost 6 h for all batches. However, using a high coriander oil level (1.90%) significantly decreased the apparent viscosity and the content of monounsaturated fatty acids. The DPPH inhibition and the content of monounsaturated fatty acids increased. The electrophoresis chromatogram exhibited a high degree of proteolysis in T2 compared to the control and T1. Microbiologically, yeast, molds, and coliforms were absent in all treatments. Feeding goats on provender supplemented with a low concentration of coriander oil may positively impact the resultant milk's technological and sensorial properties.

## Introduction

Goat milk production has been on the rise globally, as it is considered a healthier alternative to cow's milk due to its unique chemical composition. In 2021, global goat milk production was estimated at 20.4 million metric tons, with Asia and Africa being the most prominent producers^1^. The Middle East and North Africa (MENA) region is also a significant producer of goat milk, with Egypt being one of the leading countries in the region. In Egypt, goat milk production has been increasing steadily, with an estimated 215,000 metric tons produced in 2018. Small-scale farmers produce most goat milk in Egypt, mainly consumed locally, with some exports to neighboring countries^2^.

Goat milk differs from cow's milk in chemical composition, as it has higher levels of some essential nutrients, like calcium, phosphorus, and vitamin B12; it also has a higher proportion of medium-chain fatty acids, which are more easily digested by the body than the long-chain fatty acids found in cow's milk^3^. Additionally, goat milk has lower levels of lactose and a different protein structure than cow's milk, as it has a lower casein concentration than cow's milk and almost no αs1-casein making it more tolerable for people with lactose intolerance or cow's milk protein allergy^4^. Due to its unique composition, goat milk has several health benefits, including improved digestion, bone health, and a more robust immune system. It is also used to produce various dairy products, such as cheese, yogurt, and ice cream, adding to its versatility and market demand.

Recently, there has been an interest in using essential oils as feed additives for lactating animals due to their potential health and productivity benefits. Essential oils are natural compounds extracted from various plants and have been shown to possess antimicrobial, antioxidant, and immunomodulatory properties^5^. Among the essential oils studied as potential feed additives for lactating animals is coriander oil, derived from the Coriandrum sativum plant in the Apiaceae family. Coriander oil contains several bioactive compounds, including linalool, α-pinene, and terpinene^6^.

Several studies have demonstrated coriander oil's antimicrobial and antioxidant properties, indicating its potential as a natural alternative for various infectious diseases and oxidative stress-related conditions^6,7^. Additionally, coriander oil has been found to possess anti-inflammatory effects by suppressing inflammatory mediators and reducing inflammatory markers^7^. Its potential therapeutic use in metabolic and gastrointestinal disorders is suggested by its positive effects on blood glucose levels and digestive function^8^.

In a recent study conducted by Sriranga et al.^9^, the effects of dietary supplementation with coriander oil on milk production, composition, and antioxidant status were evaluated in dairy cows. The results showed that cows fed coriander oil had significantly higher milk yield and fat content than the control group. Moreover, coriander oil supplementation improved the antioxidant status of the cows, as evidenced by a significant increase in the activity of antioxidant enzymes. Other essential oils, including oregano, cinnamon, and peppermint, have also been studied as feed additives for lactating animals^10^. However, it is worth noting that the efficacy of essential oils as feed additives may vary depending on the animal species, the composition of the oil, and the dosage used.

Rayeb milk is a traditional dairy product consumed for centuries in various countries worldwide, including Egypt and the Middle East, as a refreshing drink or condiment for various dishes^11^. It is a fermented dairy product made from fresh milk left to sour naturally and known for its unique flavor, thick consistency, and various health benefits. The production process of rayeb milk involves the addition of lactic acid bacteria, which ferment the lactose in the milk, producing lactic acid and other compounds that give Rayeb milk its characteristic taste and texture^12^.

Adding probiotic cultures during the rayeb production process produces so-called bio rayeb, which enhances bio rayeb properties as health benefits by promoting the growth of beneficial intestinal bacteria. They have shown numerous health benefits, including improving gut health, reducing inflammation, and boosting the immune system^13^. Also, Hamad et al.^14^ revealed that adding probiotics and tamr to goat bio rayeb milk can improve its nutritional value and, sensory quality, overall acceptability, also significantly increase the acidity, protein content, and probiotic bacteria counts of bio rayeb, making it a more convenient and accessible probiotic product for consumers. Combining essential oils and traditional dairy products, such as rayeb milk, presents a promising approach to improving dairy products' nutritional and health benefits. The potential benefits of using essential oils as feed additives for lactating animals, combined with the health-promoting properties of traditional dairy products, could enhance the functionality and marketability of these products. Thus, this study aims to evaluate the effect of coriander oil as a feed additive on the production, composition, and sensory properties of goat bio rayeb milk, providing insight into the potential use of essential oils to enhance the nutritional value and sensory quality of traditional dairy products.

## Material and methods

### Materials

This study collected raw goat milk without any interaction with the herd from an experimental farm at the Faculty of Agriculture, Alexandria University, Egypt, after a previous study by Kholif et al.^15^ involving three goat groups. The control group (C) received the basal diet without a supplement, while two other groups were fed with 0.95 g (low coriander treatment; T1) and 1.9 g (high coriander treatment; T2) of crude coriander oil per kg DM feed daily. The crude coriander oil supplement was added to the feed twice daily to ensure the total consumption of the recommended dosage. The Direct Vat Set (DVS) of *Lactobacillus delbrueckii* ssp*. bulgaricus* and *Streptococcus salivarius* ssp*. thermophilus* in a ratio of 1:1 was obtained from Chr. Hansen's Lab in Copenhagen, Denmark.

### Methods

To manufacture the bio rayeb, three samples were prepared using raw goat milk from the three groups of goats mentioned above. The three samples were C, T1, and T2, corresponding to the control group, low coriander treatment, and high coriander treatment, respectively. Standardized milk with 3.5% fat was heated at 90 ˚C for five minutes, cooled to 41 °C, and inoculated with 3% of the yogurt culture (The Direct Vat Set (DVS) of *Lactobacillus delbrueckii* ssp. *bulgaricus* and *Streptococcus salivarius* ssp. thermophilus in a ratio of 1:1), and incubated at 42 °C until complete coagulation. The coagulated milk was blended for 5 min and refrigerated at 4 °C. The three samples of rayeb milk were analyzed on the first day and after 21 days. The manufacturing process of bio rayeb followed the method described by^16^.

### Chemical and physiochemical analysis

The titratable acidity content of samples was determined according to the Association of Official Analytical Chemists (AOAC)^17^. The pH value was measured electrometrically in all samples using a glass electrode type digital (Model, Crison-Basic 20-EU) pH meter according to the method by^18^. The conventional Gerber's Method determined the fat content as described by^19^. The total protein content of Rayeb was determined by the semi-micro Kjeldahl as described by^19^. Apparent viscosity was measured in fresh products and, at 7, 14, and 21 days of cold storage, was determined using a Bohlin coaxial cylinder viscometer (Bohlin Instrument Inc., Sweden) attached to a workstation loaded with V88 viscometer programming software. The viscometer probe, system C30, was placed in the yogurt sample cup, and viscosity measurements were carried out at 20 ± 2 °C^20^.

### Phenolic, flavonoid content, and antioxidant potentials

Preparing yogurt supernatant to determine radical scavenging activity and total phenolic content, ten g of yogurt rayeb samples were centrifuged at 4330×*g* for 5 min at 4 °C. Then, the supernatants were re-centrifuged under the same conditions and stored at − 80 °C until use. The Total Phenolic Content (TPC) expressed as a gallic acid equivalent in the µg/g sample was determined by the Folin– Ciocalteu method. Total Flavonoid Content (TFC) was assessed via the colorimetric method described by^21^. The results were expressed as µg of catechol equivalent per g of sample. The 2,2-diphenyl-1-picrylhydrazyl (DPPH) assay was performed as described by^22^. Antioxidant activity was expressed as IC50 (mg mL^−1^), where the inhibition percent of the DPPH radical was 50%.

### Fatty acids composition

#### Fat extraction and derivatization

The fat was extracted from raw and fermented milk using a modified chloroform–methanol extraction method developed by Tapia, A.M. et al.^23^. According to Radwan et al.^24^, fatty acid methyl esters were produced using sulphuric-methanol.

### GC–MS Analysis

Gas chromatography–mass spectrometry (GC–MS) analysis was performed with a TRACE GC Ultra Gas Chromatograph (THERMO Scientific Corp., USA) linked to a Thermo mass spectrometer detector (ISQ Single Quadrupole Mass Spectrometer). The GC–MS system utilized a TR-5 MS column with a 30 m 0.32 mm inner diameter and 0.25 m film thickness. Using helium as the carrier gas at a flow rate of 1.0 mL/min and a split ratio of 1:10, the following temperature program was implemented: 60 °C for 1 min, 240 °C at a rate of 4 °C per minute, and 1 min at 240 °C. The injector and detector were maintained at 210 °C. Continuous injections of 1 L diluted samples (1:10 hexane, v/v) of the mixes were performed. Electron ionization (EI) at 70 eV acquired mass spectra with a spectral range of m/z 40–450. The chemical ingredients of the essential oil were identified using AMDIS software (www.amdis.net) and retention indices (relative to n-alkanes C8–C22), as well as mass spectrum matching legitimate standards (when available), the Wiley spectral library collection, and the NSIT library database. The proportion of specific fatty acids was determined by dividing their peak area by the overall peak area of detected acids multiplied by 100.

### Electrophoresis

SDS-PAGE, 12.5% T, was conducted under reducing conditions using the discontinuous buffer system described by^25^. SDS-PAGE was performed on bio rayeb samples using a Mini-PROTEAN electrophoresis cell (Bio-Rad Laboratories, Hercules, CA, USA). One ml of each Rayeb sample was stirred with 1 mL of sample buffer for 10 min. Samples were denatured by boiling for 5 min, and then 7 µL of each sample was injected.

### Microbiological analysis

Appropriate serial dilutions of bio rayeb samples were made to obtain the bacterial count using 2% sodium citrate and enumerate using the pouring plate method. For the enumeration of *S. thermophilus*, counts were performed on M17 agar (Biolife, Italy) and incubated at 37 °C for 48 h. The *L. bulgaricus* was performed on MRS agar (Biolife, Italy) and incubated aerobically at 37 °C for 72 h^26^. The enumeration of yeasts and molds was performed as recommended by^27^ using potato dextrose agar (Difico, Italy) acidified with 10% tartaric acid and incubated at 25 °C for 5 days. Violet red bile lactose agar (Oxide, UK) was used for the coliform count, according to^28^. The plates were incubated at 37 °C for 24 h. The colony-forming units were measured as Log10 CFU g^−1^.

### Sensorial analysis

The sensory evaluation of the bio rayeb samples was carried out according to^29^. The scores used were 5 points for flavor, 4 points for body and texture, and 1 point for appearance, with an overall score of 10.

### Statistical analysis

All samples were withdrawn twice with triplicate analysis. Results were analyzed by analysis of variance (ANOVA) and Duncan's multiple mean comparisons^30^. The level of significance was (p ≤ 0.05), and all statistical analyses were performed using SPSS statistical software (version 16.0; SPSS Inc., Chicago, IL, USA).

## Results and discussion

### Physiochemical analysis

**Figure 1:**
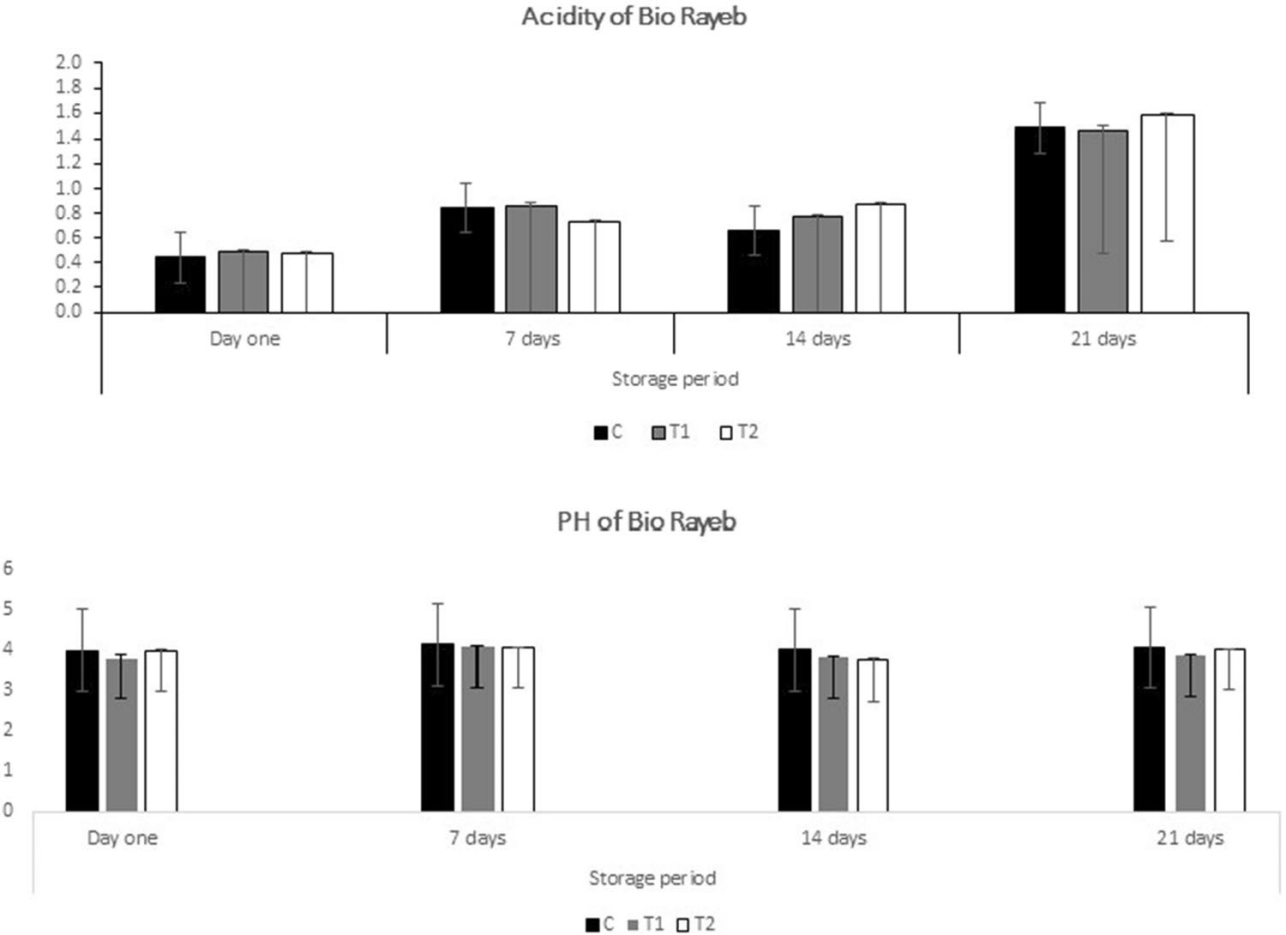
Acidity and pH of bio rayeb.

The effects of coriander oil on the time it took for Rayeb to coagulate and its pH and titratable acidity after 21 days in the refrigerator were analyzed physiochemically. Rayeb's coagulation time analysis revealed that adding coriander oil had no discernible effect. After 6 h, the pH of each batch reached 4.6, consistent with previous findings^31^. The changes in pH and titratable acidity values of Rayeb treatments during 21 days of cold storage are depicted in Fig. 1. Around pH 4.0, treatments had no significant differences on the first day of storage. The pH values of all treatments decreased significantly (p 0.05) after 21 days of storage, ranging from 4.0 to 3.87. After the storage period, T2 had the lowest pH level (3.87). These outcomes are similar to those reported in^32^. Intriguingly, the opposite trend was observed in the titratable acidity of all treatments, which gradually increased over time. T2 had significantly (p 0.05) higher titratable acidity (1.58) than other treatments. These results indicate that adding coriander oil did not significantly alter the pH and titratable acidity levels of Rayeb. Adding coriander oil to goat feed had no significant effect on the coagulation time or pH and titratable acidity values of bio rayeb. These results can provide valuable information to the dairy industry regarding using coriander oil as a potential feed additive with a positive effect on resultant milk. Further investigation into the effects of coriander oil on other physiochemical parameters of rayeb and other dairy products can be conducted.

### Chemical composition

Table 1 illustrates the effect of crude coriander oil supplementation in goat feed on the chemical composition of bio rayeb, specifically its fat, total protein, lactose, and total solids contents. The results indicate that the chemical composition of bio rayeb remains within the normal range, and the measured values for these properties are consistent with those reported by earlier researchers ^26,33^**.** These findings contribute to the existing knowledge and provide additional insight into the potential benefits of crude coriander oil as a goat feed supplement.

**Table 1:** Chemical composition of bio rayeb.

Treatments						
Parameter	Day one			21 days		
	C	T1	T2	C	T1	T2
Fat	3.50^a^ ± 0.1	3.49^a^ ± 0.2	3.50^a^ ± 0.4	3.2^b^ ± 0.4	3.0^b^ ± 0.5	2.92^c^ ± 0.6
Protein	3.34f. ± 0.1	3.94^e^ ± 0.1	4.18^c^ ± 0.1	4.36^b^ ± 0.1	4.14^d^ ± 0.1	4.82^a^ ± 0.2
Lactose	3.13^d^ ± 0.2	4.12^b^ ± 0.2	4.42^a^ ± 0.2	3.03^e^ ± 0.2	3.52^c^ ± 0.2	3.90^b^ ± 0.2
TS	10.58f. ± 0.4	11.88^c^ ± 0.5	12.52^a^ ± 0.6	11.01^e^ ± 0.5	11.31^d^ ± 0.7	12.27^b^ ± 0.6
Ash	0.61^d^ ± 0.03	0.62^d^ ± 0.03	0.62^d^ ± 0.03	0.62^c^ ± 0.04	0.65^a^ ± 0.03	0.63^b^ ± 0.04

Each reported value is the mean ± SD of three replicates. Means in the same row followed by different letters are significantly different (*p* < 0.05).

### Phenolic, flavonoid content, and antioxidant potentials

The antioxidant activity was quantified in fresh bio rayeb samples. The data obtained are presented in Table 2. Bio rayeb with high coriander oil concentration T2 was more affluent in the ratio of DPPH inhibition than the control. This was due to the effect of coriander oil supplementation. These results were consistent with Delgado-Pertíñez et al.^34^, who confirmed that replacing cereal with dry orange paper in the diet of goats during the early lactation phase had no significant effect on flavonoid content. However, the resultant milk's α-tocopherol, total phenolic compound (TPC), and total antioxidant capacity (TAC) increased.

**Table 2:** Antioxidant activity (DPPH and ABTS radical scavenging assay) of Bio Rayeb.

Time	Treatment	(DPPH) IC50 (µl/ml)	(ABTS) IC50 (µg/ml)	Total phenolic content (µg GAE/g)
	Ascorbic acid	11.16 ± 0.43	11.60 ± 1.49	–
Day one	C	134.23 ± 0.92^d^	146.60 ± 0.85^b^	204.02 ± 5.16^a^
	T1	224.91 ± 1.65^b^	100.69 ± 1.13f.	146.87 ± 4.42^d^
	T2	287.28 ± 0.49^a^	160.53 ± 0. 50^a^	150.17 ± 6.37^d^
21 days	C	131.33 ± 1.15^e^	143.33 ± 0.57^c^	194.82 ± 0.78^b^
	T1	130.95 ± 0.93^e^	116.28 ± 1.45^d^	165.34 ± 9.98^c^
	T2	182.57 ± 0.51^c^	113.85 ± 0.79^e^	173.54 ± 5.98^c^

IC50: effective concentration, which achieves 50% DPPH radical scavenging activity. ABTS· + radical assay expressed as percentage activity as a function of the concentration of bio rayeb, GAE/g: phenolic content expressed as milligrams of gallic acid equivalents per gram. Each reported value is the mean ± SE of three replicates. Means in the same column followed by different letters are significantly different (p < 0.05).

### Fatty acid composition

Table 3 displays the findings of the gas-chromatography (GC) analysis of the fatty acid composition of bio rayeb at the onset of storage and the end of storage. The table illustrates the content of short-chain fatty acids (SCFAs), long-chain fatty acids (LCFAs), polyunsaturated fatty acids (PUFAs), and monounsaturated fatty acids (MUFAs). Additionally, the table indicates the statistical significance (S or NS) of the distinctions in fatty acid content between the raw and fermented milk at the start and end of storage.

**Table 3:** Gas–liquid chromatographic analysis of bio rayeb fatty acids content at day one and end of storage.

Treatments	Symbol	C			T1			T2		
Type		Raw milk	Bio rayeb	*Sig*	raw milk	Bio rayeb	*Sig*	Raw milk	Bio rayeb	*Sig*
Fatty acids										
SFAs										
Butyric acid	C4:0	4.99^a^	0.23^b^	*S*	0.47	0.53	*NS*	0.43	0.63	*NS*
Caproic acid (goaty)	C6:0	2.09^a^	0.24^b^	*S*	1.02	1.06	*NS*	3.05	1.12	*NS*
Caprylic acid	C8:0	2.14^b^	11.3^a^	*S*	2.31	2.15	*NS*	2.42	2.81	*NS*
Capric acid	C10:0	3.96^b^	29.0^a^	*S*	8.18	6.74	*NS*	5.81	10.4	*NS*
Undecanoic acid	C11:1	11.9^a^	0.21^b^	*S*	2.26	0.12	*NS*	10.9^a^	0.19^b^	*S*
Lauric acid	C12:0	1.27^b^	10.8^a^	*S*	3.01	2.68	*NS*	3.16	4.53	*NS*
Tridecanoic acid	C13:0	1.31	0.53	*NS*	0.20	0.13	*NS*	1.84	0.26	*NS*
Myristic acid	C14:0	4.41	3.19	*NS*	9.14	8.76	*NS*	9.00	11.6	*NS*
Pentadecanic acid	C15:0	0.56	1.69	*NS*	1.33	1.44	*NS*	0.76	1.44	*NS*
Palmitic acid	C16:0	32.1	28.7	*NS*	27.6	25.7	*NS*	21.5^b^	28.8^a^	*S*
Heptadecanic acid	C17:0	0.85	1.00	*NS*	1.07	1.07	*NS*	0.76	1.22	*NS*
Stearic acid	C18:0	10.5^a^	3.09^b^	*S*	12.3^a^	2.52^b^	*S*	9.03	3.66	*NS*
Arachidic acid	C20:0	0.77	0.67	*NS*	0.34	0.32	*NS*	0.32	0.18	*NS*
Behanic acid	C22:0	1.54	ND	*-*	0.32	ND	*-*	0.79	0.17	*NS*
PUFA’s										
Myristoleic acid	C14:1	0.04	ND	*-*	0.09	0.05	*NS*	0.14	0.11	*NS*
Heptadecanoic acid	C17:1	0.15	0.26	*NS*	0.21	0.37	*NS*	0.29	0.38	*NS*
Linoleic acid	C18:2 n-6	1.27^b^	3.21^a^	*S*	2.88	3.17	*NS*	2.90	3.94	*NS*
Linolelaidic acid	C18:2 t/n-6	0.17	0.21	*NS*	0.19	0.14	*NS*	0.24	0.15	*NS*
Arachdonic acid	C20:4 n-6	0.52	ND	*-*	0.23	0.25	*NS*	0.04^b^	0.67^a^	*S*
Erucic acid	C22:1 n-9	0.22	0.15	*NS*	ND	0.01	*-*	0.02	ND	*–*
MUFA’s										
Palmitoleic acid	C16:1 n-7	0.89	1.22	*NS*	1.24	1.99	*NS*	0.48^b^	1.93^a^	*S*
Oleic acid	C18:1 n-9	12.4	18.3	*NS*	21.0	26.3	*NS*	22.1	20.2	*NS*
Summary of the essential fatty acid parameters of fat extracted from Treatments										
SFAs		75.6	90.5	*NS*	69.4^a^	53.3^b^	*S*	69.4	67.0	*NS*
UFAs		15.5	23.3	*NS*	25.7	32.3	*NS*	25.9	27.4	*NS*
MUFAs		13.7	19.9	*NS*	22.5	28.7	*NS*	22.8	22.6	*NS*
PUFAs		1.82	3.42	*NS*	3.25	3.57	*NS*	3.09^b^	4.77^a^	*S*
PUFAs:MUFAs ratio		13.7	17.0	*NS*	14.5	12.4	*NS*	13.6^b^	21.1^a^	*S*
UFAs:SFAs ratio		21.2	25.7	*NS*	37.3^b^	60.6^a^	***S***	37.4	41.2	*NS*

*SFAs* saturated fatty acids, *UFAs* unsaturated fatty acids, *MUFAs* monounsaturated fatty acids, *PUFAs* polyunsaturated fatty acids, *Sig* significance, *S* significance, *NS* non-significance, *ND* non-detected.

Twenty-two fatty acids were identified in both the raw milk and bio rayeb treatments after 14 days of storage, comprising 14 saturated short, medium, and long chains (C4–C18), two monounsaturated medium and long chains (C16 and C18), and six polyunsaturated long-chains (C18:C22), as shown in Table 3.

Palmitic oil (C16:0) was all treatments' most prevalent fatty acid^35^. The control raw goat milk had the highest percentage of C16:0, accounting for 32.1%, whereas the rate decreased in bio rayeb treatments, where T1 recorded the lowest value (25.7%). On the other hand, the content of MUFAs such as palmitoleic acid (C16:1 n-7) increased in rayeb treatments compared to raw milk.

Furthermore, the results indicate that fermentation led to significant changes in the fatty acid content of bio rayeb. For instance, the content of capric acid (C10:0) considerably increased from 3.96% in raw milk to 29.0% in fermented milk. In contrast, butyric acid content (C4:0) reduced significantly from 4.99% in raw milk to 0.23% in fermented milk. The increase in capric acid content could result from the lactic acid bacteria (LAB) used for fermentation, which can convert C10:0 to capric acid^36^. The decrease in butyric acid content may be due to the utilization of butyric acid by the LAB during fermentation or by producing volatile compounds responsible for the flavor and aroma of fermented milk^37^.

Oleic acid (C18:1 n-9) followed the same pattern, with its content being higher in bio rayeb treatments than in raw milk and the T1 bio rayeb having the highest value (26.3). Moreover, the content of polyunsaturated fatty acid conjugated linoleic acid (C18:2 n-6) also increased in bio rayeb samples compared to raw milk treatments. Conjugated linoleic acid (CLA) is an essential precursor that reduces or eliminates cancer, prevents heart disease, enhances immune function, and plays a vital role in treating obesity or building lean body mass^38^.

According to Chilliard et al.^39^, the proportion of acetate and alpha-hydroxybutyrate, essential precursors to the synthesis de novo of short- and medium-chain fatty acids (SMCFA) in the mammary gland, increases when cows consume more forage. Using 1.9% coriander oil in the goat feeding resulted in a higher content of saturated fatty acids in the T2 raw milk (C6 and C14). Bio rayeb control and T1 treatments showed no significant changes in the content of MUFAs and PUFAs. Additionally, the UFAs: SFAs ratio was significant in bio rayeb T1 (60.6%) compared to all treatments, and UFAs displayed the highest values in the T2 bio rayeb compared to the other treatments. As mentioned earlier, the supplementation of goat feed with coriander oil positively impacted the proportion of conjugated linoleic acid (CLA) and unsaturated fatty acids in bio Rayeb, making it a healthier product for consumers.

### Electrophoresis

**Figure 2:**
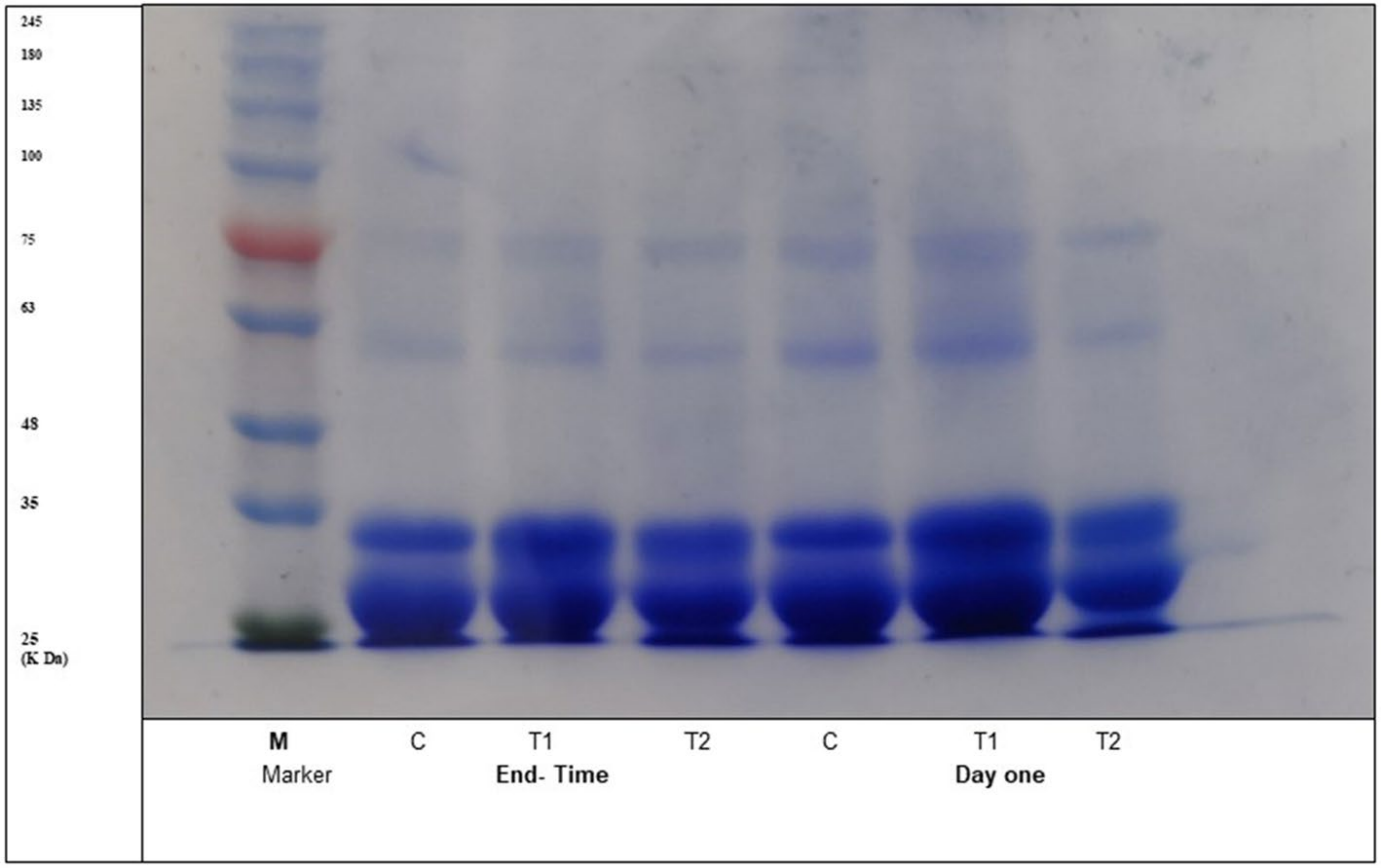
Original sodium dodecyl sulfate–polyacrylamide gel electrophoresis (SDS-PAGE; 12.5%T) chromatogram of Bio rayeb at day one and end of storage.

In Fig. 2, the electrophoretogram analysis of bio rayeb on day one and after 21 days of cold storage revealed prominent αs2-Casein bands, with little to no presence of αs1-Casein. This outcome was unsurprising given that goat milk has a lower concentration of αs1-Casein than αs2-Casein, as opposed to cow milk^40^. Interestingly, T2 bio rayeb exhibited higher proteolysis than the control and T1, and this may be attributed to the activation of the starter culture by coriander oil, increasing its count. These findings are consistent with a previous study investigating the variations in protein composition in fermented milk during cold storage^41^.

### Microbiological analysis

**Figure 3:**
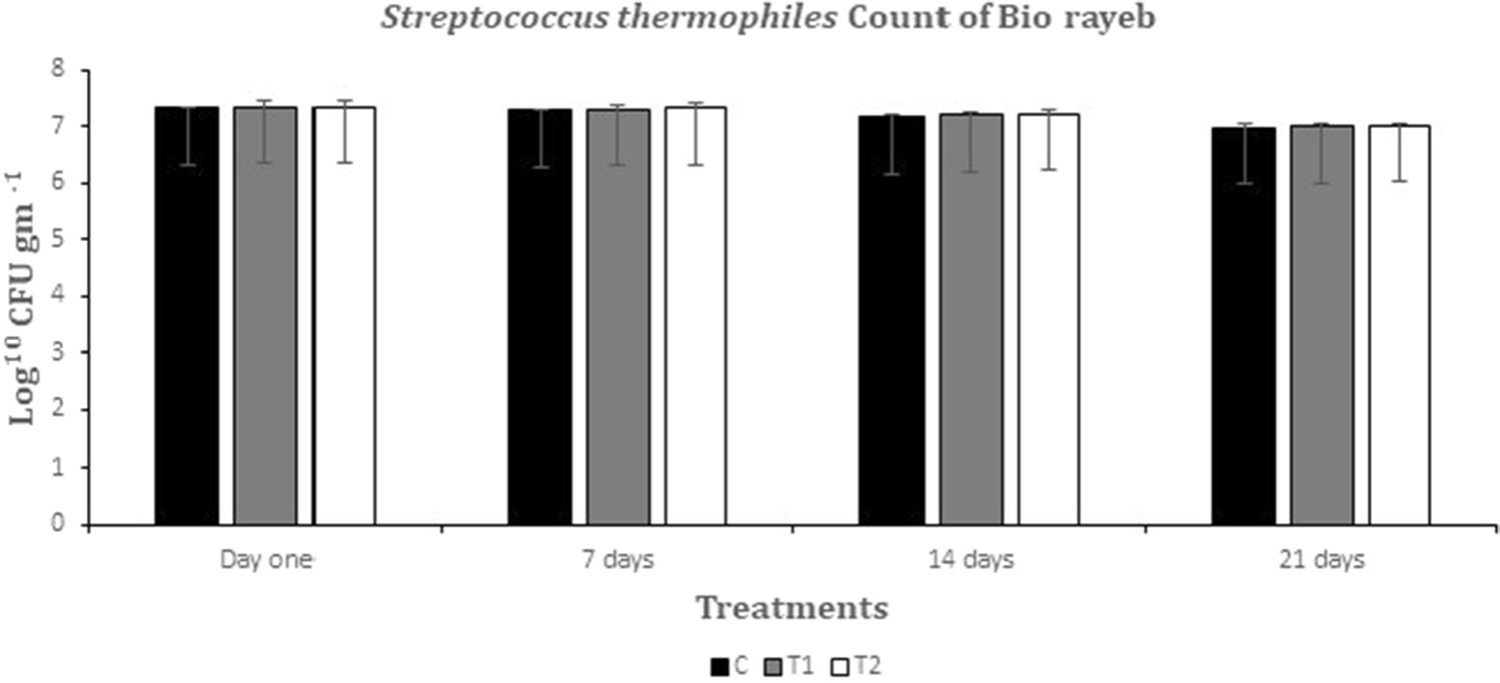
*Streptococcus thermophiles* growth during the storage period.

**Figure 4:**
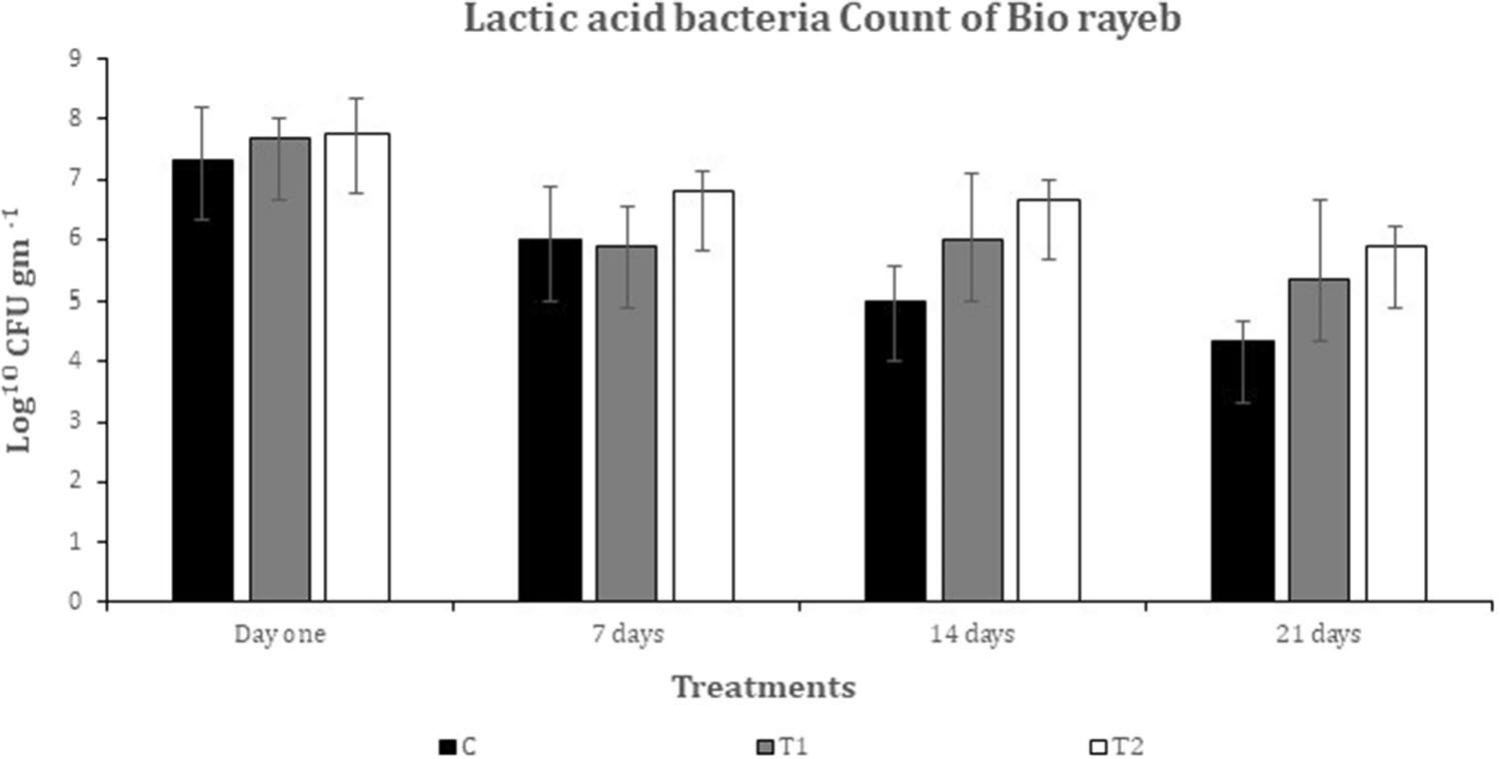
Lactic acid bacteria growth during the storage period.

Figures 3 and 4 depict the alterations in the viable counts of *S. thermophiles* and *L. bulgaricus* resulting from various Rayeb treatments. The quantities of *S. thermophilus* and *L. bulgaricus* in T1 and T2 were comparable to the control, as observed in^42^. These findings were consistent with the aforementioned study. However, the *L. bulgaricus* count in bio rayeb T1 and T2 was higher than that of the control due to coriander oil's stimulation of microbial growth, which agreed with the results of^43^. Titratable acidity data confirmed these results, with higher values in T1 and T2, as depicted in Fig. 1, supporting the microbiological analysis. Moreover, the counts of S. thermophilus and *L. bulgaricus* exhibited insignificant declines during storage. No yeast, molds, or coliforms were present, which could be attributed to the excellent hygienic conditions maintained throughout the manufacturing process.

### Apparent viscosity

Table 4 displays the apparent viscosity of bio rayeb on days one, 7, 14, and 21 of storage. Bio rayeb is known for its intricate shear-thickening and time-dependent flow behavior, vital for quality, product development, and handling^44,45^. The findings indicate a substantial rise in rayeb shear-thickening for all treatments at reduced shear rates. The inclusion of varying levels of coriander oil considerably (p ≤ 0.05) boosted the apparent viscosity of bio rayeb during cold storage compared to the control, and this may be due to the increased number or strength of coagulated gel particles or better particle hydration, as noted in^46^.

**Table 4:** Apparent viscosity of Bio Rayeb.

Viscosity (cP)				
Treatment	Day one	7 days	14 days	21 days
C	276.8^d^ ± 2.0	281.9^c^ ± 4.9	283.9^ab^ ± 1.5	293.0 ^a^ ± 2.9
T1	113.7^b^ ± 1.1	114.3^b^ ± 0.8	115.1^b^ ± 1.7	186.6 ^a^ ± 1.2
T2	108.7^b^ ± 2.6	114.2^b^ ± 1.1	114.8^b^ ± 1.9	114.9^b^ ± 1.9

Each reported value is the mean ± SD of three replicates. Means in the same row followed by different letters are significantly different (*p* < 0.05), *cP* centipoise.

### Sensorial properties

**Figure 5:**
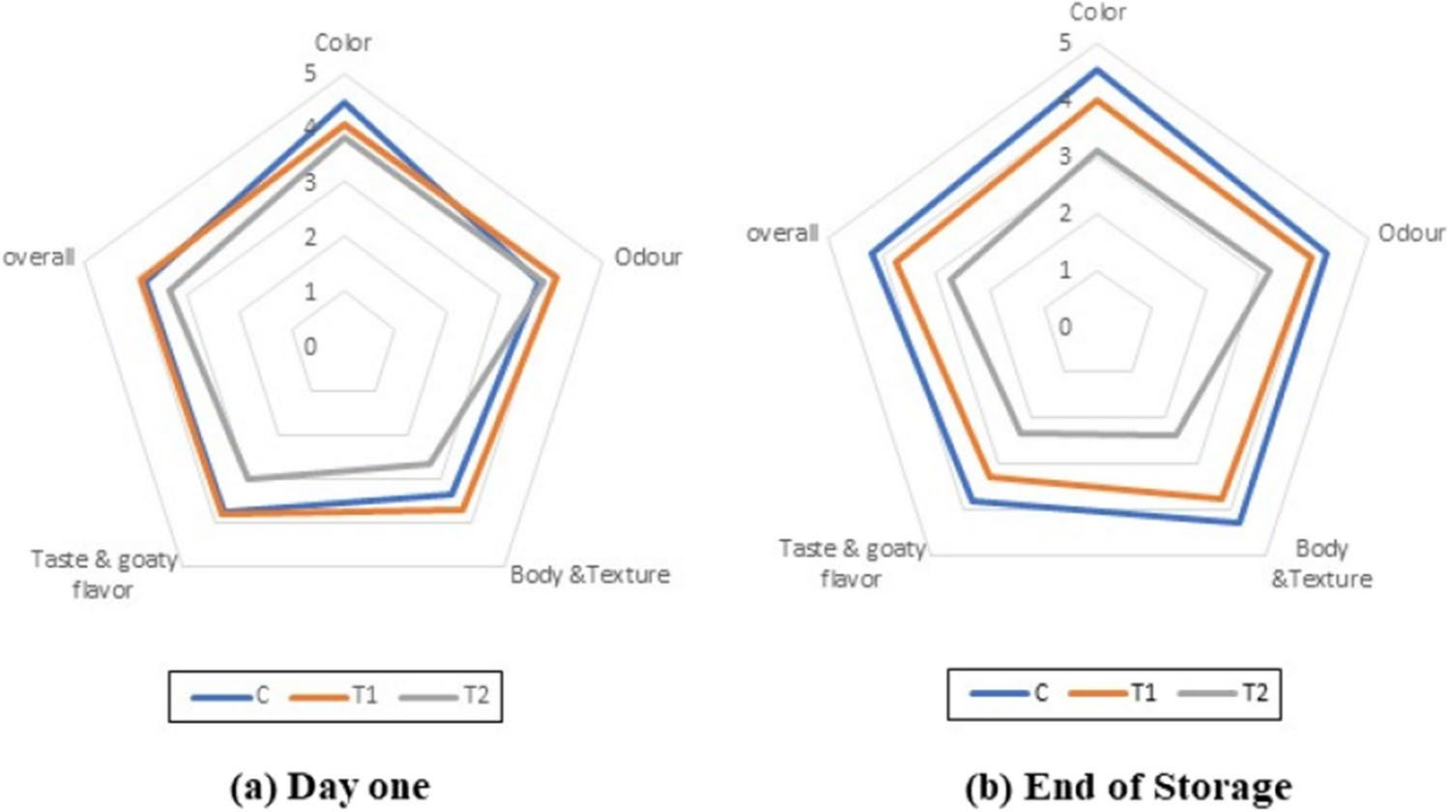
The sensory evaluation of Bio rayeb treatments (**a**; day one and **b**; the end of storage).

Figure 5 (a: day one and b: end of storage) depicts the sensory properties of bio rayeb treatments compared to the control on day one and after refrigerated storage. The panelists favored treatment T1 over the control, while T2 received the lowest acceptability scores, particularly concerning body and texture. Interestingly, no changes were observed in sensory parameters or acceptability after 21 days of refrigerated storage. These results suggest that the most suitable level of coriander oil to incorporate into goat feed to obtain acceptable sensory properties in the final product, including color, odor, taste, and goaty flavor, is 0.95%. These findings are attributed to the positive impact of certain coriander oil fatty acids, such as linalool fatty acid, which is the primary component of Coriandrum sativum seed oil (64.4%)^47^. These fatty acids have a pleasant floral flavor that modifies the goaty taste. Shariati et al.^48^ also reported similar findings, where dough containing 0.05% coriander leaf extract was preferred by panelists and received the highest scores.

## Conclusion

In summary, the physicochemical analysis of bio rayeb revealed that the addition of coriander oil did not significantly affect its coagulation time, pH, or titratable acidity levels. However, the titratable acidity levels gradually increased, with T2 displaying the highest level. The chemical composition of bio rayeb remained consistent with earlier studies, with normal levels of fat, total protein, lactose, and total solids content.

Moreover, the study investigated the antioxidant potential, phenolic, and flavonoid content of bio rayeb and found that T2 had a higher DPPH inhibition ratio than the control, indicating a positive effect of coriander oil supplementation on bio rayeb's antioxidant activity. The fatty acid composition analysis showed significant changes due to fermentation, with increased capric acid and decreased butyric acid content. Additionally, bio rayeb samples showed increased oleic acid and polyunsaturated fatty acid conjugated linoleic acid compared to raw milk treatments.

Furthermore, the sensory analysis demonstrated that adding coriander oil to goat feed improved the sensory properties of bio rayeb. The electrophoretogram analysis revealed a higher proteolysis rate in T2, likely due to coriander oil's activation of the starter culture. Finally, the apparent viscosity of bio rayeb significantly increased with the addition of coriander oil during cold storage. These findings provide valuable insights for the dairy industry to enhance goat milk products' nutritional and functional properties, with the potential for increased consumer appeal.

## Supplementary Information

Supplementary Information.

## Data Availability

The datasets used and/or analyzed during the current study are available from the corresponding author upon reasonable request.
