# Supplementary material for: Impact of mixing coriander oil with goat feed on the chemical, microbiological and sensory characterizations of bio rayeb milk
Source: Sci Rep. 2023 Jul 11;13:11215. doi: 10.1038/s41598-023-38047-3 (PMC10336081; doi:10.1038/s41598-023-38047-3)

**This is the original, unprocessed version of the gel
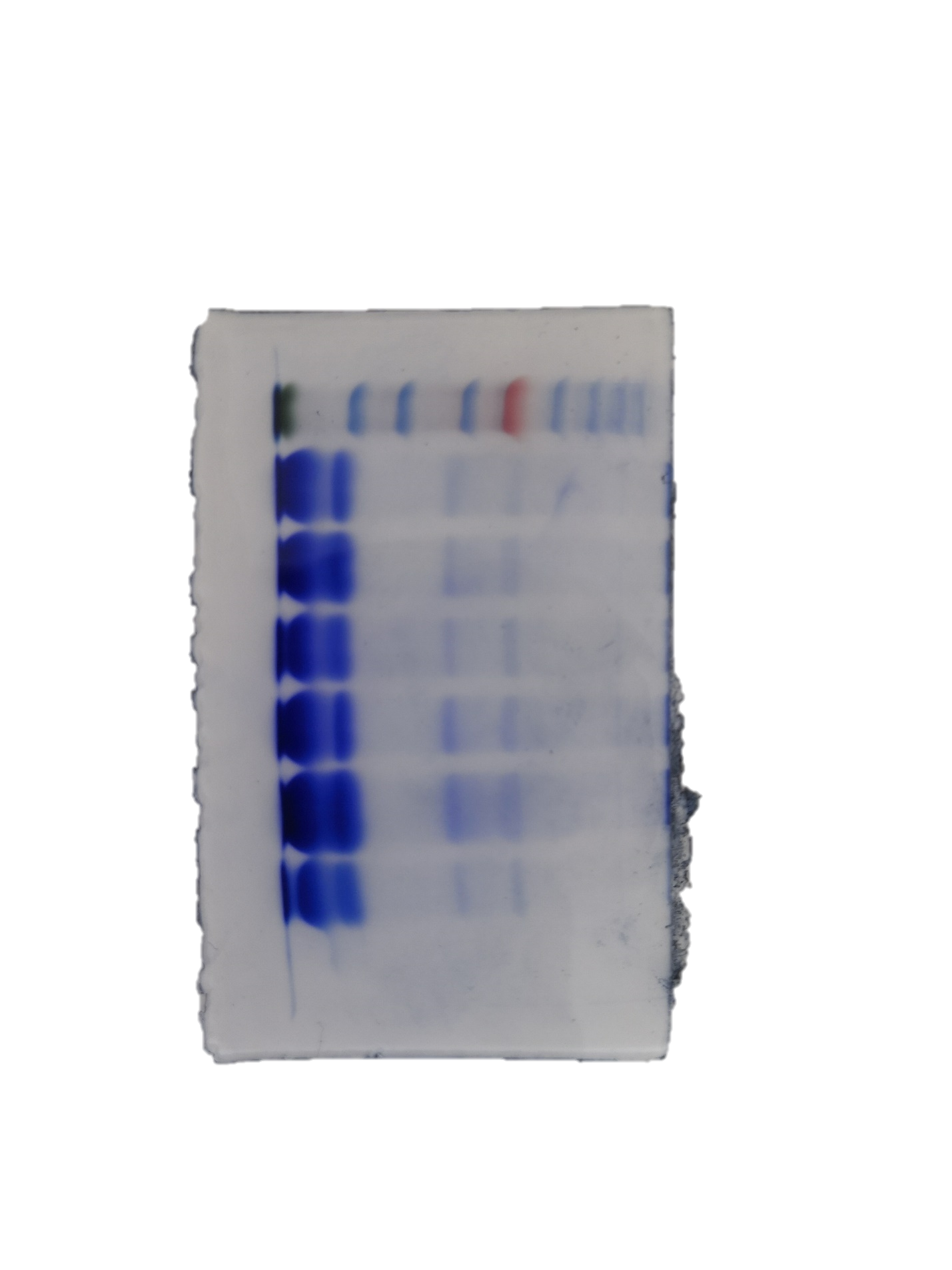
 image with its original length and dimensions**; The method used was SDS-PAGE, 12.5% T, which was conducted under reducing conditions using the discontinuous buffer system described by **lameli 1970**. SDS-PAGE was performed on bio rayeb samples **using a Mini-PROTEAN electrophoresis cell (Bio-Rad Laboratories, Hercules, CA, USA).** One ml of each Rayeb sample was stirred with 1 mL of sample buffer for 10 min. Samples were denatured by boiling for 5 min, and then 7 µL of each sample was injected.

The edited version declares the image information


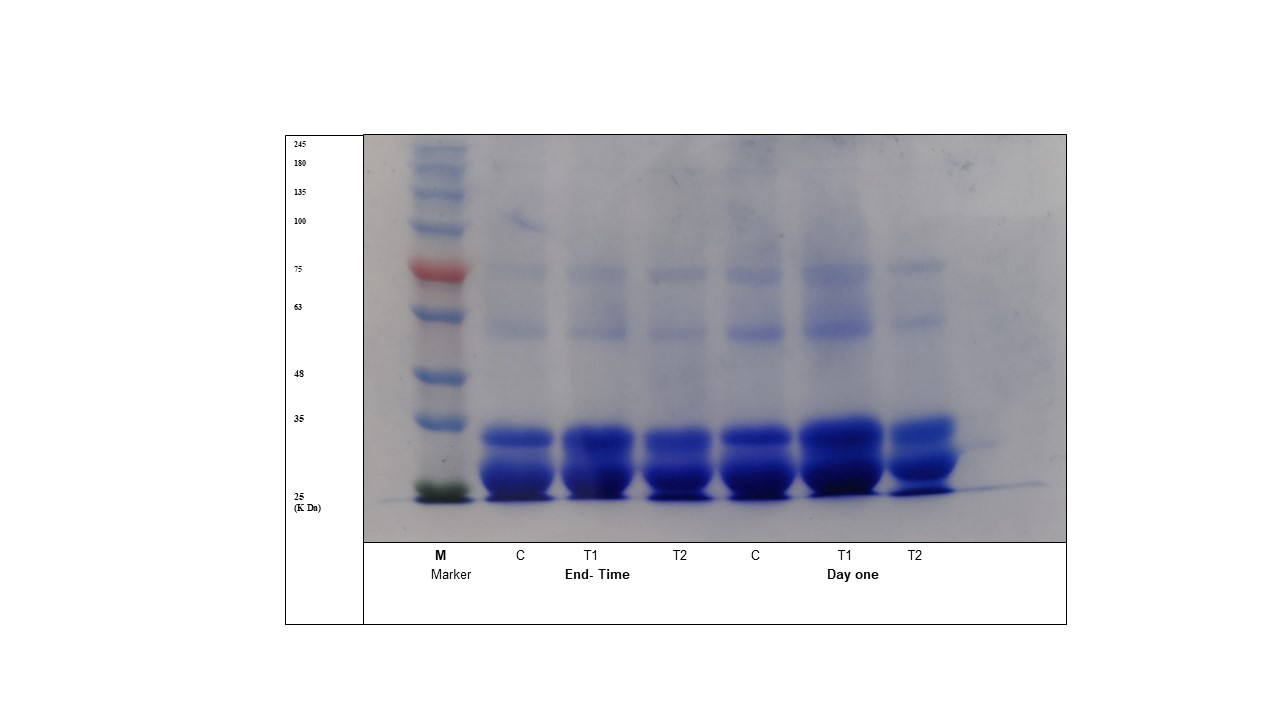

Supplement: Supplementary file 1 — Supplementary Information. [file 41598_2023_38047_MOESM1_ESM.docx]
